# Prevalence and Visual Consequences of Non-adherent Patients Receiving Anti-vascular Endothelial Growth Factor (VEGF) Injections at King Fahad Specialist Hospital (KFSH), Qassim Region

**DOI:** 10.7759/cureus.44340

**Published:** 2023-08-29

**Authors:** Abdulmajeed D Alharbi, Noura I Alotayk, Abdulmajeed A Alaboudi, Abdulrahman y Alammar, Mohammad I Aldekhail, Meshari A Alharbi, Thekra A Alsamel, Muhannad A Aljutayli, Ayman M Aljarbou, Osama M Aljameeli

**Affiliations:** 1 Department of Ophthalmology, Qassim University, Qassim, SAU; 2 College of Medicine, Qassim University, Buraydah, SAU; 3 Department of Ophthalmology, King Fahad Specialist Hospital (KFSH), Buraydah, SAU

**Keywords:** visual consequences, retinal diseases, missed appointment, dme, anti-vegf injection

## Abstract

Background: Anti-vascular endothelial growth factor (VEGF) injection treatment is a widely utilized therapy for various retinal diseases, including diabetic macular edema (DME). Therefore, the importance of compliance and follow-up should be discussed with the patient. There have been no studies conducted in the Qassim region to estimate the prevalence of patients missing their anti-VEGF appointments. To fulfill this need, we conducted this study to evaluate the compliance rate of patients treated with anti-VEGF injections for DME as well as to determine the visual consequences of the delay in anti-VEGF treatment in the Qassim region.

Methodology: This observational retrospective cohort study was conducted at King Fahad Specialist Hospital (KFSH) in the Qassim region of Saudi Arabia. We extracted all file numbers of patients who were using intravitreal anti-VEGF injections to treat DME. The data were managed and analyzed using the IBM Statistical Package for the Social Sciences (SPSS) software (IBM Corp., Armonk, NY, USA).

Results: In the current study, we were able to collect data from 198 patients who received anti-VEGF treatment in the hospital. Among the participants, 57.6% were male, with a mean age of 61.7 years old (standard deviation (SD) = 10.23). Among the patients, we found that the rate of non-adherence to the anti-VEGF injection was 54.5%, and those patients delayed their scheduled doses for more than 56 days. In 47.5% of the patients, delaying or stopping their appointments had no known reason; however, blepharitis was the main reason for delaying or stopping the dose in 27.7% of the patients, followed by endophthalmitis in 18.7% of the patients. There is no significant difference between before and after stopping the treatment, considering visual acuity (VA) or optical coherence tomography (OCT). However, regarding the disease progression, we found that 15 out of the 30 patients had worsened in the OCT after they missed their injections (mean increase in the VA was 6.069 (SD = 97.45), t = -0.278, P = 0.783, and decrease in the OCT was -14.9667 (SD = 133.87, P = 0.454).

Conclusion: There is a high rate of patients who missed their appointments for an anti-VEGF injection. This resulted in the worsening of OCT in half of the 30 patients who were enrolled in the visual consequences study, which had a negative impact on treatment and disease progression.

## Introduction

In diabetes mellitus (DM), insulin secretion or action is impaired, causing blood sugar levels to rise [1]. Diabetic retinopathy (DR) is a major complication in diabetic patients that causes vision loss. After 20 years of diabetes, nearly all patients with type 1 and 60% of patients with type 2 diabetes develop diabetic retinopathy [2]. One of the primary factors in the development of DR is the pathological release of vascular endothelial growth factor (VEGF). In cases of DR, however, VEGF release results in pathological angiogenesis that is irregularly distributed and features poorly constructed vessels that are prone to leak, leading to fluid buildup within the retina [3]. In late 2004, a series of anti-VEGF treatments such as pegaptanib and ranibizumab were granted FDA approval to treat DR based on successful large, multicenter clinical trials [3]. Intravitreal injections of anti-VEGF agents work by blocking the growth of abnormal new blood vessels in the retina. It can prevent vision loss and, in some instances, improve vision. Still, it needs compliance to be more effective [4].

One study has shown that patients who did not comply with intravitreal anti-VEGF therapy for DME had considerably worse visual results than those who did [5]. The probability of having a proliferative DR was also 13 times higher in patients with a loss of follow-up [5]. Strategies for improving compliance and adherence to medicine should be taken into consideration for the best possible patient care when considering the risk of disease progression [5].

Sobolewska B et al. have found that missing appointments is significantly common, ranging from 39% to 100% of patients [6]. They arranged the patients in this study into three groups based on data for different treatment periods: treatment period for Group 1 < 30 months; treatment period for Group 2 > 30 months; and treatment period for Group 3 > 60 months [6]. In the third group with a treatment period > 60 months, all the patients had a treatment gap of more than 56 days [6].

A recent global study recruited 30,138 patients; there were 4,710 DME patients in the study, and 1,063 were treatment-naïve [7]. Saudi Arabia was the country with the most treatment-naïve patients with DME (6.4%) [7]. At baseline, the mean age was 64.5 years; 54.7% were male, and 69.2% were White. At one year, the mean visual acuity (VA) letter score improved by +3.5 (n = 502) from a baseline of 57.7 with a mean of 4.5 injections. Presented by injection frequencies ≤4 and ≥5, VA letter score gains were 0.5 (n = 264) and 6.9 (n = 238) from baseline letter scores of 56.6 and 59.0, respectively. Over five years, the incidence of ocular and non-ocular serious adverse events was 0.3% and 5.8%, respectively. No endophthalmitis cases were reported [7].

Our aim in this study is to determine the prevalence of patients missing their anti-VEGF appointments at King Fahad Specialist Hospital (KFSH) during 2021-2022, in the Qassim region of Saudi Arabia. Additionally, we are looking for the visual consequences of the delay in anti-VEGF injection.

## Materials and methods

This was an observational retrospective cohort study that was conducted among patients receiving anti-VEGF injections for DME at KFSH in the Qassim region of Saudi Arabia in the period between 2021 and 2022.

All files of DME patients receiving anti-VEGF treatment at KFSH were selected for the study. Patients with diabetic macular edema were included in the study. Patients receiving anti-VEGF for other causes (e.g., central retinal vein occlusion (CRVO), age-related macular degeneration (AMD)) and patients who missed the anti-VEGF treatment because they required retinal surgery due to complications of diabetic retinopathy were excluded from the study.

We extracted all file numbers of patients who were suffering from DME. Then, we checked the compliance of the patients and looked for patients who postponed or missed their appointment for anti-VEGF treatment for 56 days (eight weeks) or more. Those who missed their appointments for 56 days or more were classified as non-adherent, whereas those who missed them for less than 56 days were classified as continuously adherent. The definition of non-adherence had very different variations in the literature, including the extent of irregular or absent visits that exceeded the four-week follow-up by more than two weeks, and some studies reported more than 60 days between visits and others for eight weeks or more [8]. In the current study, as reported, we defined non-adherence as missing treatment for 56 days or more. However, for the visual consequences, we extracted 30 random files from the non-adherent patients who came again for investigation and collected their VA and optical coherence tomography (OCT) before and after missing doses. All patients who met the exclusion criteria were dropped.

The data collected were anonymized, and no patient identifiers were used, ensuring confidentiality. The study was conducted after receiving ethical permission from the Qassim region’s research ethics committee (approval number: 607-44-20). After collecting the data, we reviewed the prevalence of patients missing their anti-VEGF appointments during 2021-2022. Variables were coded before being analyzed using IBM Statistical Package for the Social Sciences (SPSS) software, version 26 (IBM Corp., Armonk, NY, USA). Simple percentages (%) were used to present the results. Statistical significance is defined as a probability level (p-value) of 0.05 or less.

## Results

In the current study, we were able to collect data from 198 patients who received anti-VEGF at KFSH. Among the participants, 57.6% of them were male, with a mean age of 61.7 years old (standard deviation (SD) = 10.23), whereas 57.1% of the patients were older than 60 years. In addition, 92.9% of the patients postponed their treatment for more than one month or stopped the treatment, with 75.5% of them stopping or delaying their doses after the first dose and 24.5% after the second dose. In total, the mean number of days missing a dose among the patients was 156.5 days (SD = 147.2), while among the adherent group, the mean delay was 33.63 days, and in the non-adherent group, the mean number of days missing was 216.25 days (Table 1).

**Table 1:** Demographic factors of the participants (N=198) and the mean number of days missing an anti-VGEF dose VGEF: vascular endothelial growth factor

		Count	Column N%
Gender	Male	114	57.6%
	Female	84	42.4%
Age (in years)	18–30	3	1.5%
	31–45	6	3.0%
	46–60	76	38.4%
	> 60	113	57.1%
	Mean (SD)	61.70 (10.23)	
Delayed or stopped after dose	One dose	139	75.5%
	Two doses	45	24.5%
Days missing dose mean (SD)	Total	156.5 (147.20)	
	Adherent group	33.63 (21.23)	
	Non-adherent group	216.25 (155.47)	

**Figure 1:**
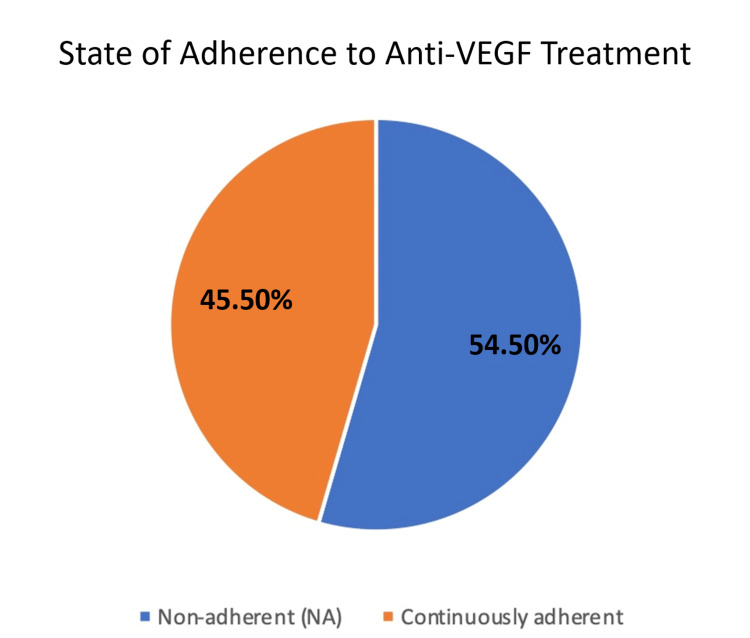
State of adherence to anti-VEGF treatment VGEF: vascular endothelial growth factor

Among the patients, we found that the rate of non-adherence to the anti-VEGF treatment was 54.5%, where those patients delayed their doses for more than 56 days, while good adherence was reported among 45.5% of the patients, where 18 out of 90 adherent patients showed no delays (0 days) to their appointment (Figure 1).

**Figure 2:**
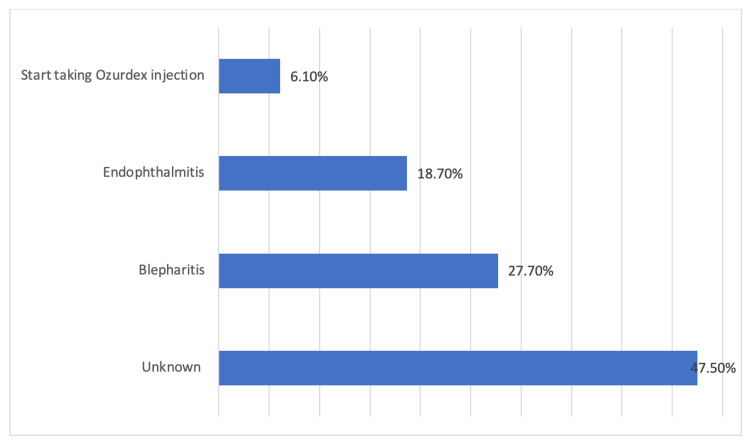
Reasons for the delay or stopping of the anti-VEGF injection dose VGEF: vascular endothelial growth factor

In almost 47.5% of the patients, delaying or stopping their appointments had no known reason; however, blepharitis was the main reason for delaying or stopping the dose in 27.7% of the patients, followed by endophthalmitis in 18.7% of the patients (Figure 2).

In this study, neither the gender of the patients nor their age had a significant impact on the level of adherence to anti-VEGF appointments (P = 0.420 and 0.884, respectively). However, it seems that female patients showed a slightly higher tendency to stop the treatment than male patients (58.3% vs. 51.8% of male patients were non-adherent). Moreover, it was found that there is no significant difference between patients with different age groups considering adherence to anti-VEGF appointments. However, we noticed a tendency to be more adherent in older age groups > 60 years old in comparison to the 46-60 year age group considering adherence to anti-VEGF appointments (Table 2).

**Table 2:** Gender and age-wise comparison in regard to adherence to anti-VGEF appointments VGEF: vascular endothelial growth factor

		Non-adherent (NA) or continuously adherent				
		Non-adherent (NA)		Continuously adherent		P-value
		Count	Row N %	Count	Row N %	
Gender	Male	59	51.8%	55	48.2%	0.420
	Female	49	58.3%	35	41.7%	
Age (in years)	18–30	1	33.3%	2	66.7%	0.884
	31–45	3	50.0%	3	50.0%	
	46–60	42	55.3%	34	44.7%	
	> 60	62	54.9%	51	45.1%	

For assessment of the consequences of missing doses of the anti-VEGF injection, 30 patients were included, of whom 46.2% were treated for the left eye, 38.5% for the right eye, and 15.4% for both eyes. Moreover, 23.3% of them stopped taking doses after dose one, 73.3% after dose two, and one case after dose three (out of six). Before stopping the treatment, the mean VA in the affected eye was 20/125.75, ranging from 20/20 to 20/400 (SD = 20/118.1); however, after stopping the doses, the mean VA was 20/127.48 (SD = 119.18), which ranged from 20/20 to 20/400, with no significant difference between before and after stopping the treatment (the mean increase in the VA was 6.069 (SD = 97.45), t = -0.278, P = 0.783). In addition, the mean OCT before stopping the treatment was 425.733 (SD = 129.8), ranging between 188 and 695, which was reduced to 410.766 (SD = 160.38) after stopping the treatment; however, there was no significant difference between before and after stopping the treatment (P = 0.545) (Table 3).

**Table 3:** Consequences of missing doses of anti-VEGF injection representing VA and OCT in the last test and after the missing dose, and the difference between them VGEF: vascular endothelial growth factor; VA: visual acuity; OCT: optical coherence tomography

	N	Minimum	Maximum	Mean	Std. Deviation
VA: previous result (right or left eye): 20/	30	20.00/ 20.00	20.00/ 400.00	20.00/ 125.7500	20.00/ 118.10404
VA after the missed dose (right or left eye): 20/	30	20.00/ 20.00	20.00/ 400.00	20.00/ 127.4828	20.00/ 119.18701
Difference in VA (after the missed dose)	30	20.00/ -200.00	20.00/ 200.00	20.00/ 6.0690	20.00/ 97.45656
OCT: previous result (right or left eye)	30	188.00	695.00	425.7333	129.80434
OCT after the missed dose (right or left eye)	30	138.00	830.00	410.7667	160.38911
Difference in OCT after the missed dose	30	-467.00	267.00	-14.9667	133.87294

## Discussion

Anti-vascular endothelial growth factor medications administered intravitreally have revolutionized the treatment of retinal diseases, including DME. Ranibizumab, an approved treatment, was introduced to the market as a consequence of successful phase III randomized controlled studies in which elderly patients with retinopathy were examined every month for 24 months [9-10]. A consistent improvement in eyesight was not achieved in other clinical trials that involved quarterly hospital visits. [11-12]. There are still questions about the long-term effects of ranibizumab medication and how well clinical practice results can mimic those found in clinical trials [13-14]. To our knowledge, there have not been any reports of both the incidence of missed hospital appointments and the impact of such missed hospital appointments on patients in Saudi Arabia, despite the significance of missed hospital appointments in this context and their potential to affect the long-term outcomes of therapy for retinal illnesses, including DME. Therefore, the aim of the current study was to determine the prevalence of patients missing their anti-VEGF appointments at King Fahad Specialist Hospital during 2021-2022, in the Qassim region of Saudi Arabia.

In the current study, the rate of non-adherence to the anti-VEGF treatment was 54.5%; those patients delayed their doses for more than 56 days, while good compliance was reported among 45.5% of the patients. This is in agreement with the results of previous studies, where, according to the Inhibition of VEGF in Age-related Choroidal Neovascularisation (IVAN) trial, the authors showed that 35% of the patients had at least one missed hospital appointment [15]. Real-world studies show that patients with retinal diseases, including DME, have considerable losses to follow-up in comparison to clinical trials, which often show good patient adherence to visits (DME). According to Obeid et al.’s research, 22.2% (2,003 of 9,007) of retinal disease patients receiving intravitreal anti-VEGF injections had at least one 12-month period of lost follow-up over a four-year period [16]. A further study by Obeid et al. revealed a comparable percentage of attrition in those with proliferative diabetic retinopathy (PDR): 28% (356 of 1,272) of PDR patients who had had panretinal photocoagulation (PRP) and 22.1% (228 of 1,030) of PDR patients receiving intravitreal injections lost follow-up for more than a year, respectively [17]. Gao et al. also discovered that 25.3% (413 out of 1,632) of patients with non-proliferative DR and DME missed their scheduled appointment visits for more than a year [18]. However, those studies reported a lower non-adherence rate; other studies reported a similar or higher rate of non-adherence, including a safety-net hospital trial. Sixty-one percent of patients with PRP were lost to follow-up for more than six months [19]. A retrospective cohort of PDR patients in Houston, Texas, revealed a total loss of follow-up rate of 52.4% and interval loss of follow-up rates of 17.7% for six months and 10.6% for 12 months for patients who returned to care after a significant delay [20]. Patients with retinopathy experience significant rates of loss of follow-up globally as well. French researchers Boulanger-Scemama et al. investigated treatment non-adherence in neovascular age-related macular degeneration (nAMD) patients on ranibizumab. During the trial period, the overall loss of follow-up (patients who never came back) was 57% [21]. According to Kelkar et al. from India, patients with nAMD and those who had DME experienced follow-up loss rates of 33.5% and 38%, respectively [22]. These studies’ various loss of follow-up definitions, study designs, and study populations may all be contributing factors to differences in loss of follow-up rates. Regardless of location, loss of follow-up is a global public health issue that may have an irreversible impact on individuals with diabetic retinopathy and diabetic retinopathy’s visual results, resulting in a lower quality of life as well as higher societal expenditures.

Individuals with a visual impairment may find it difficult to do everyday tasks or be unable to work, necessitating the need for friends, relatives, or other social help, which is more expensive. Patients with diabetic retinopathy experience negative visual outcomes when their medication is stopped or interrupted. In the current study, we did not find a significant difference between before and after the stopping of treatment, but VA was worsened by a mean difference of 6.07, which indicates the negative impact of stopping the treatment on VA. However, it is important to mention that the number of missing days from the last dose to the follow-up is very variable among the patients; therefore, this result could reduce the significance of the results. Despite this limitation, our results were similar to the results of previous studies in the literature. In comparison to our results, a previous study conducted by Kim et al. showed that nAMD patients receiving anti-VEGF therapy who discontinued their treatment course despite intraretinal fluid exhibited significant deterioration in VA (approximately six lines of vision) by 24 months of follow-up. After 24 months of follow-up, Kim et al. showed that nAMD patients receiving anti-VEGF medication who quit their treatment course despite intraretinal fluid exhibit considerable VA degradation (about six lines of vision) [23]. Soares et al. also looked into the functional and anatomic effects of losing track of patients with nAMD. Following their return, 93 eyes with a minimum six-month loss of follow-up were assessed. From 20/80 during the visit just before the loss of follow-up to 20/200 at the subsequent visit, the median VA dramatically deteriorated. After returning, the anti-VEGF injection course was resumed. Despite commencing therapy, VA did not reach the level before the loss of follow-up [24].

Understanding and resolving the underlying causes of loss of follow-up has become a key objective in the treatment of nAMD and DR due to the irreversible vision loss brought on by periods of loss of follow-up. Low-income status, non-White race or ethnicity, greater distance from the clinic, and poor baseline eyesight are risk factors for loss of follow-up [16-19]. Many other interventions have been proposed. In order to encourage African American patients over the age of 65 to have dilated fundus examinations, Ellish et al. showed that newsletters that were personalized for each patient or targeted to a specific patient subgroup were equally beneficial [25]. Also, it has been demonstrated that care adherence is increased by telephone-based reminder and tracking systems, regardless of socioeconomic background, gender, or spoken language [26-27]. Walker et al. conducted a randomized controlled experiment, and they found that the rate of DR screening increased by 74% when compared to the control group (P =.0005) [26]. In comparison to a written reminder given before the subsequent visit, Anderson et al. discovered that follow-up phone calls from clinic staff considerably increased the return rate following a missed ophthalmology appointment. In this study, the personalized follow-up phone call group saw a return rate of 66% compared to the conventional follow-up group’s return rate of 35% (P =.001) [28].

Limitations

Our research has had some limitations that may influence or impact its results. These limitations include about 60% of the patients; we still couldn’t figure out why they didn’t complete their anti-VEGF course. This impacted our research because we could not identify the cause in the majority of cases that led to this non-compliance. Knowing the exact cause for every patient and having continuous communication with non-compliant patients can help identify the problems that they may encounter and lead to improved compliance. We faced other limitations involving the visual outcome; most patients don’t have previously recorded OCT readings in the hospital system, and that, unfortunately, led to excluding a huge number of patients and shortening the sample size.

Recommendations

The goal is to increase awareness of DME among patients with diabetes, who should be educated early about how to manage their diabetes to slow the disease’s progression and avoid complications that could affect their vision. It could be done through campaigns directed at the community. Also, we could give the patients flexible appointments to avoid non-compliance. Furthermore, blepharitis was found to be the most common reason for postponing injections. Thus, ophthalmologists should emphasize the importance of lid hygiene to avoid blepharitis. In addition, future research should assess the anterior segment to elicit detailed information if there is another accompanying disease, like cataract. The worsening of VA could be caused by an advancement in cataracts due to long-term loss of follow-up and not taking injections. Eventually, it is recommended to save any OCT reading for future studies to overcome the problem of the small sample size.

## Conclusions

In conclusion, there is a high rate of patients who missed their appointments for an anti-VEGF injection, which showed a negative impact on the treatment and disease progression in OCT in half of the 30 patients who were enrolled in the visual consequences study. For this reason, educating the patients about the importance of completing their treatment regimen and the negative consequences of delaying or losing follow-up would have a great effect on reducing the rate of missed appointments.
